# Deep Learning for Automatic Subclassification of Gastric Carcinoma Using Whole-Slide Histopathology Images

**DOI:** 10.3390/cancers13153811

**Published:** 2021-07-29

**Authors:** Hyun-Jong Jang, In-Hye Song, Sung-Hak Lee

**Affiliations:** 1 Catholic Big Data Integration Center, Department of Physiology, College of Medicine, The Catholic University of Korea, Seoul 06591, Korea; hjjang@catholic.ac.kr; 2 Department of Hospital Pathology, Seoul St. Mary’s Hospital, College of Medicine, The Catholic University of Korea, Seoul 06591, Korea; ihsongih@catholic.ac.kr

**Keywords:** gastric cancer, differentiated, undifferentiated, mucinous, deep learning, digital pathology

## Abstract

**Simple Summary:**

The histopathologic type is one of the most important prognostic factors in gastric cancer (GC), which underpins the basic strategy for surgical management. In the present study, a fully automated approach was applied to distinguish differentiated/undifferentiated and non-mucinous/mucinous tumor types in GC tissue whole-slide images from The Cancer Genome Atlas (TCGA) stomach adenocarcinoma dataset (TCGA-STAD). The patch-level areas under the curves for the receiver operating characteristic curves for the differentiated/undifferentiated and non-mucinous/mucinous classifiers were 0.932 and 0.979, respectively. We also validated the classifiers on our own datasets and confirmed that the generalizability of the classifiers is excellent. The results indicate that the deep-learning-based tissue classifier could be a useful tool for the quantitative analysis of cancer tissue slides.

**Abstract:**

Histomorphologic types of gastric cancer (GC) have significant prognostic values that should be considered during treatment planning. Because the thorough quantitative review of a tissue slide is a laborious task for pathologists, deep learning (DL) can be a useful tool to support pathologic workflow. In the present study, a fully automated approach was applied to distinguish differentiated/undifferentiated and non-mucinous/mucinous tumor types in GC tissue whole-slide images from The Cancer Genome Atlas (TCGA) stomach adenocarcinoma dataset (TCGA-STAD). By classifying small patches of tissue images into differentiated/undifferentiated and non-mucinous/mucinous tumor tissues, the relative proportion of GC tissue subtypes can be easily quantified. Furthermore, the distribution of different tissue subtypes can be clearly visualized. The patch-level areas under the curves for the receiver operating characteristic curves for the differentiated/undifferentiated and non-mucinous/mucinous classifiers were 0.932 and 0.979, respectively. We also validated the classifiers on our own GC datasets and confirmed that the generalizability of the classifiers is excellent. The results indicate that the DL-based tissue classifier could be a useful tool for the quantitative analysis of cancer tissue slides. By combining DL-based classifiers for various molecular and morphologic variations in tissue slides, the heterogeneity of tumor tissues can be unveiled more efficiently.

## 1. Introduction

Gastric cancer (GC) is generally classified into two-tiered categories according to several official classification systems [1,2,3]. The Japanese classification categorizes GC as differentiated and undifferentiated types according to the degree of glandular differentiation [1]. The former includes papillary adenocarcinoma and well- to moderately differentiated tubular adenocarcinoma, whereas the latter includes poorly differentiated adenocarcinoma and signet ring cell carcinoma. For mucinous adenocarcinoma cases, the specimen is considered an undifferentiated type, even if the GC originated from the differentiated component [1,4]. Furthermore, poorly differentiated carcinomas are subclassified into solid or non-solid types. Solid-type GCs display solid structures and barely recognizable tubules, whereas non-solid-type tumors consist of individual cells or clusters of a few cells with diffuse infiltrative growth patterns [5].

The histomorphologic type is one of the major prognostic factors in GC, which underpins the basic strategy for surgical management. It is reported that the undifferentiated carcinomas carry a higher risk of lymph node metastasis (LNM) than differentiated types in early gastric cancer (EGC) [6,7]. Moreover, there are several studies on the clinical outcomes of mixed-type EGC, which is defined as GC that has both differentiated and undifferentiated components. Recent reports demonstrated the relationship between mixed-type histology and higher risk of LNM in EGC [8,9,10,11]. Mucinous GC is also linked to a poor prognosis [12]. Therefore, identifying between different subtypes of GC is essential for decision making, especially regarding the surgical resection of EGC.

Computer-based analysis of tissue images is drawing attention as an area of research in digital pathology. Recently, deep learning (DL) has been applied to detect and classify diverse tumors, including GC [13,14,15]. DL using convolutional neural network (CNN) systems have considerable visual recognition capabilities because they can discern features directly from the large training dataset, outperforming humans [16]. With the recent approval of using the digitized pathology whole-slide images (WSIs) for primary diagnosis, the digitized tissue slides have been explosively increasing in prevalence, providing massive amounts of digital pathology images [17]. However, DL in digital histopathology is still in its early stages of development, and the efficiency and accuracy of pathologic diagnosis could be improved by combining the routine digitization of WSIs with DL.

In the present study, we built DL-based differentiated/undifferentiated and non-mucinous/mucinous GC tissue classifiers to automatically classify the GC WSIs based on The Cancer Genome Atlas (TCGA) stomach adenocarcinoma dataset (TCGA-STAD). Then, we tested the generalizability of the classifiers on our own GC tissue dataset. Here, we report the results of the classification performance of the developed classifiers.

## 2. Materials and Methods

### 2.1. Patient Cohort

From the TCGA-STAD dataset, 396 formalin-fixed paraffin-embedded (FFPE) slides from 371 patients were selected after the basic slide quality reviews. One pathologist (S.H.L.) initially annotated normal/tumor, differentiated/undifferentiated, and non-mucinous/mucinous regions of the stomach FFPE slides, and then another pathologist (I.H.S.) reviewed the annotation. We classified the tumor tissues as mixed-type when more than 30% of differentiated or undifferentiated tissues were mixed. Questionable regions were then co-reviewed for an agreed annotation. We randomly selected 90% of patients for the training group and 10% for the test group. Therefore, slides from a patient cannot be included in both the training and test groups.

For the external validation of the classification models trained with the TCGA dataset, we collected stomach cancer tissue slides from 232 patients who previously underwent surgical resection in Seoul St. Mary’s hospital between 2017 and 2019 (SSMH dataset). Approval for this study was acquired from the Institutional Review Board of the College of Medicine at the Catholic University of Korea (KC20RISI0329). The SSMH slides were also annotated for normal/tumor, differentiated/undifferentiated, and non-mucinous/mucinous regions for exact validation of the models. The summaries of the TCGA and SSMH datasets are presented in Supplementary Table S1.

### 2.2. Pathologic Diagnosis

The histologic type of GC was diagnosed according to the Japanese classification [4]. Well- and moderately differentiated tubular adenocarcinoma and papillary adenocarcinoma were classified as differentiated-type, and poorly differentiated adenocarcinoma (either solid or non-solid types) and signet ring cell carcinoma as undifferentiated-type. In this study, mucinous adenocarcinoma was classified separately. The decision regarding the histologic classification was made by the consensus of two expert gastrointestinal pathologists (S.H.L. and I.H.S.).

### 2.3. Deep Learning Model

**Figure 1:**
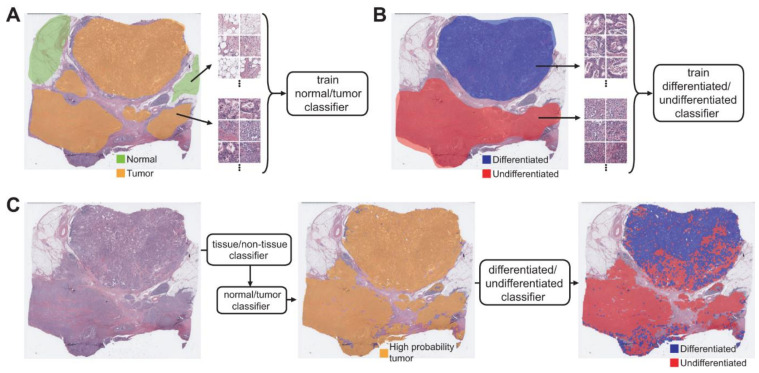
Approach for the fully automated classification of gastric cancer tissue subclasses. (**A**) A normal/tumor classifier was trained with normal/tumor tissue patches collected based on the pathologists’ annotation. (**B**) A differentiated/undifferentiated classifier was trained with differentiated/undifferentiated tumor tissue patches collected based on the pathologists’ annotation. (**C**) Sequential application of the tissue/non-tissue, normal/tumor, and differentiated/undifferentiated classifiers could automatically delineate the differentiated and undifferentiated tumor tissues.

Artifacts in tissue slides such as air bubbles, blurring, compression artifacts, pen markings, and tissue folding are irrelevant for the tissue classification tasks and thus should be removed to enhance the performance of the classifiers. We adopted a convolutional neural network (CNN)-based tissue/non-tissue classifier to select the proper tissue patches, which was used in our previous studies [18,19]. The detailed structure of the tissue/non-tissue classifier was described there. Because the discrimination of differentiated and undifferentiated tissues in this study is meaningful only for the cancer tissues, we tried to build the differentiated/undifferentiated classifier for the selected tumor tissues. Therefore, a normal/tumor tissue classifier for the TCGA FFPE stomach tissue slides is a prerequisite. In a previous study, we built normal/tumor classifiers for tissue slides of cancers from bladder, lung, colon and rectum, stomach, bile duct, and liver [20]. In the present study, we reused the normal/tumor classifiers for the stomach cancer tissues. The classifier was trained to classify the 360×360 pixel tissue patches, obtained from WSIs of the stomach tissues at 20× magnification, into normal or tumor tissue. Normal and tumor tissue patches for the training were separated into each group based on the normal/tumor tissue annotation from pathologists (Figure 1A). The patch-level areas under the curves (AUCs) for the receiver operating characteristic (ROC) curves of the normal/tumor classifier for the TCGA-STAD FFPE stomach tissue was excellent, at 0.993 [20].

To train the differentiated/undifferentiated classifier, 360 × 360 pixel patches of differentiated and undifferentiated tissue were collected based on the annotation from the pathologists (Figure 1B). Before training, the patches underwent the normal/tumor classifier and only patches classified as tumors were included for the training. The non-mucinous/mucinous classifier was also trained with the same approach. The Inception-v3 architecture was adopted for the normal/tumor, differentiated/undifferentiated, and non-mucinous/mucinous classifiers. The classifiers were implemented using the TensorFlow DL library (http://tensorflow.org). We used a mini-batch size of 128 and the same number of normal/tumor, differentiated/undifferentiated, or non-mucinous/mucinous patches were fed into each mini-batch during the training of each classifier to yield balanced results. Data augmentation techniques were applied to the tissue patches during training, including random rotations by 90° and random horizontal or vertical flipping. To avoid the effect of stain difference, color normalization was applied. To minimize overfitting, 10% of the training slides were used as a validation dataset. The training was stopped when the accuracy of the validation data was saturated. Then, the performance of the trained classifier was evaluated on the test dataset. For the classification of slides in the test datasets, the normal/tumor classifier initially discriminated the tumor patches from the entire selection of slides. Then, only tissue patches with high tumor probability were used for differentiated/undifferentiated or non-mucinous/mucinous classification (Figure 1C).

### 2.4. Statistics

To demonstrate the performance of each classifier, the ROC curves and their AUCs for the test datasets were presented. The ROC curves for the patch-level results were calculated based on the pathologists’ annotation on the WSIs. The distribution of differentiated/undifferentiated or non-mucinous/mucinous regions was visualized as heatmaps on the tissue thumbnails. We used a permutation test with 1000 iterations to compare the differences between the ROC curves when the comparison was necessary [21]. 

## 3. Results

In the present study, we tried to build a system to discriminate subclasses of GC tissues. To this end, separate classifiers to discriminate the normal/tumor tissues, differentiated/undifferentiated tumor tissues, and non-mucinous/mucinous tumor tissues in stomach tissue slides were built to characterize stomach cancer tissues. After removing various artifacts and background with the tissue/non-tissue classifier, tumor patches with high tumor probability (tumor probability higher than 0.9) were selected (Figure 1C middle panel). Then, the differentiated/undifferentiated or non-mucinous/mucinous classifiers were applied to the tumor patches (Figure 1C right panel). The classification results were overlapped on the image of the whole tissue as a colored heatmap to clearly delineate the distribution of the different subclasses of cancer tissues.

**Figure 2:**
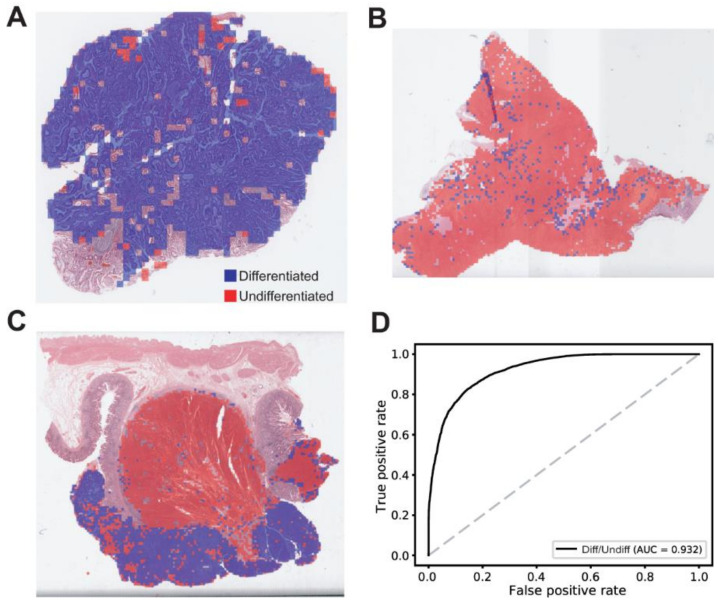
Results for the differentiated/undifferentiated classification on the TCGA-STAD dataset. (**A**) A representative heatmap overlaid on a tissue slide image demonstrating a mainly differentiated tumor tissue. (**B**) A representative heatmap overlaid on a tissue slide image demonstrating a mainly undifferentiated tumor tissue. (**C**) A representative heatmap overlaid on a tissue slide image demonstrating a mixed tumor tissue. (**D**) The patch-level area under the curve (AUC) for the receiver operating characteristic curve of the differentiated/undifferentiated classifier.

The classification results for the differentiated/undifferentiated tumor tissues are described in Figure 2. Different composition of differentiated/undifferentiated tumor tissues in a GC tissue slide was easily identifiable with the heatmap visualization: mainly differentiated tumor tissue (Figure 2A), mainly undifferentiated tumor tissue (Figure 2B), and mixed tumor tissue (Figure 2C). In comparing the classification results for the test dataset with the pathologists’ annotation, the ROC curve for the patch-level classification results could be obtained (Figure 2D). The AUC for the ROC curve was 0.932 for the differentiated/undifferentiated discrimination. The differentiated/undifferentiated classifier showed a relatively poor performance compared to the normal/tumor classifier (AUC = 0.993, *p* < 0.01 between the normal/tumor and differentiated/undifferentiated classifiers by Venkatraman’s permutation test for unpaired ROC curves). 

**Figure 3:**
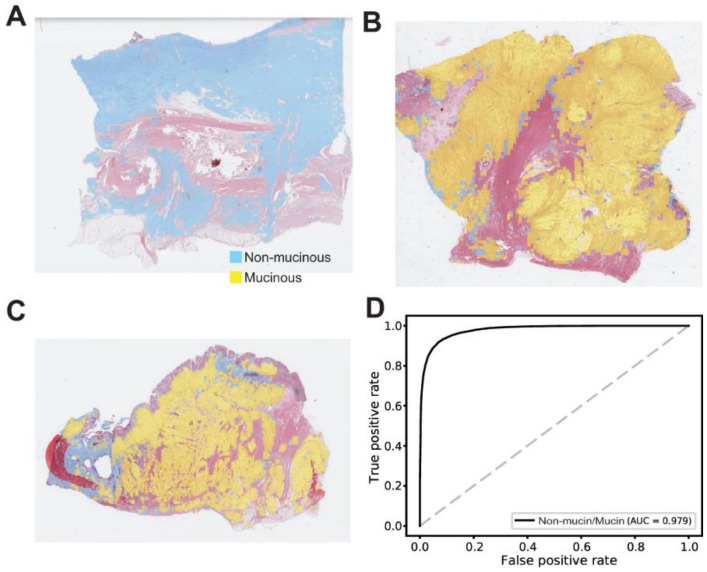
Results for the non-mucinous/mucinous classification on the TCGA-STAD dataset. (**A**) A representative heatmap overlaid on a tissue slide image demonstrating a mainly non-mucinous tumor tissue. (**B**) A representative heatmap overlaid on a tissue slide image demonstrating a mainly mucinous tumor tissue. (**C**) A representative heatmap overlaid on a tissue slide image demonstrating a relatively mixed tumor tissue. (**D**) The patch-level area under the curve for the receiver operating characteristic curve of the non-mucinous/mucinous classifier.

Non-mucinous/mucinous classification results are shown in Figure 3. There were mainly non-mucinous tumor tissues (Figure 3A), mainly mucinous tumor tissues (Figure 3B), and relatively mixed tumor tissues (Figure 3C). The AUC for the ROC curve was 0.979 for the non-mucinous/mucinous classifier. This result was better than the differentiated/undifferentiated classifier (*p* < 0.05 by Venkatraman’s permutation test for unpaired ROC curves).

**Figure 4:**
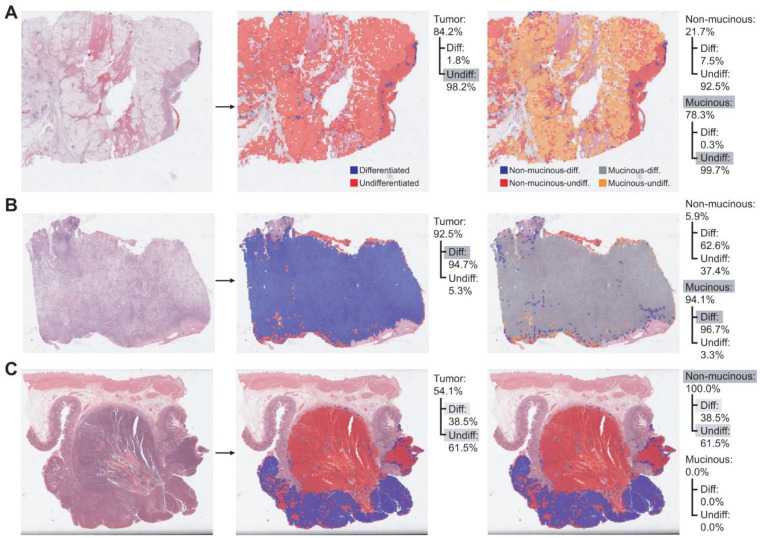
Quantitative evaluation of the subclasses in gastric cancer tissues. (**A**) A representative cancer tissue consisting of mainly undifferentiated and mucinous tissues. (**B**) A representative cancer tissue consisting of mainly differentiated and mucinous tissues. (**C**) A representative cancer tissue consisting of mixed differentiated, undifferentiated and non-mucinous tissues. The relative proportion of each subclass was calculated. Grey shades indicate the major components.

The classifiers can be used for the quantitative description of the tumor tissues (Figure 4). By applying the normal/tumor classifier, the proportion of tumor tissues in the total tissue area can be obtained. For example, there are tumor proportions of 84.2%, 92.5%, and 54.1% in the tissues of Figure 4A, B, and C, respectively. Since the total area of the tissues can be easily calculated from the resolution of the WSIs and the number of tissue patches, we can also calculate the tumor area with the tumor proportion. Then, the differentiated/undifferentiated proportion of the tumor tissue can be obtained via the differentiated/undifferentiated classifier. Their distribution in the tissues can also be easily identified with the heatmaps (Figure 4 middle panels). Finally, the non-mucinous/mucinous classifier can delineate the mucinous tumors. By combining the differentiated/undifferentiated and non-mucinous/mucinous classifiers, the non-mucinous-differentiated, non-mucinous-undifferentiated, mucinous-differentiated, mucinous-undifferentiated areas can be identified (Figure 4 right panels). Therefore, the proportion of subclasses of cancer tissues in a tissue slide can be quantitatively described and delineated with the sequential application of our tissue/non-tissue, normal/tumor, differentiated/undifferentiated, and non-mucinous/mucinous classifiers.

**Figure 5:**
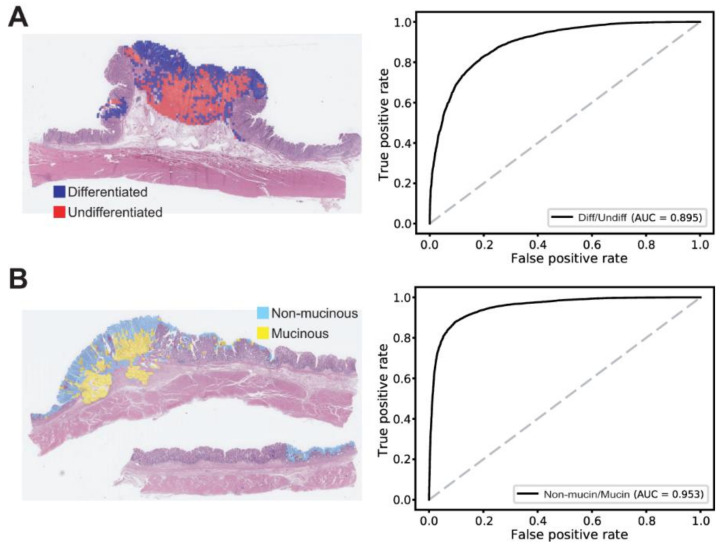
Extra validation of the classifiers trained with the TCGA dataset. (**A**) Classification results of the differentiated/undifferentiated classifier on the SSMH stomach tissues. Left panel: A representative gastric cancer tissue with mixed differentiated/undifferentiated tumor tissues. Right panel: The patch-level area under the curve (AUC) for the receiver operating characteristic (ROC) curve of the differentiated/undifferentiated classifier. (**B**) Classification results of the non-mucinous/mucinous classifier on the SSMH stomach tissues. Left panel: A representative gastric cancer tissue with mixed non-mucinous/mucinous tumor tissues. Right panel: The AUC for the ROC curve of the non-mucinous/mucinous classifier.

One important aspect of DL application is the generalizability of a model for external datasets that were not exposed during the training session. Although the test sets in the TCGA dataset were not exposed during the training, they may share specific features with the training dataset that may not be very generally applicable outside the TCGA dataset. Therefore, a completely different dataset is necessary to test the generalizability of a trained model. We tested the classifiers on the SSMH dataset. In a previous study, we showed that the normal/tumor classifier for the TCGA FFPE stomach cancer tissues showed similar performance for the SSMH dataset (AUC = 0.991) [20]. In the present study, we tested the generalizability of the TCGA-trained differentiated/undifferentiated and non-mucinous/mucinous classifiers on the SSMH dataset. The AUCs for the ROC curves for the patch-level classification results were obtained by comparing the classification results for the SSMH dataset with the pathologists’ annotation on the SSMH dataset. The AUCs were 0.895 and 0.953 for the differentiated/undifferentiated and non-mucinous/mucinous classifiers, respectively (Figure 5). The performance was comparable to the result for the TCGA test dataset (*p* = 0.119 and *p* = 0.203 for the differentiated/undifferentiated and non-mucinous/mucinous classifiers, respectively, by Venkatraman’s permutation test for unpaired ROC curves). The classification results for the TCGA and SSMH datasets are summarized in Supplementary Table S2.

**Figure 6:**
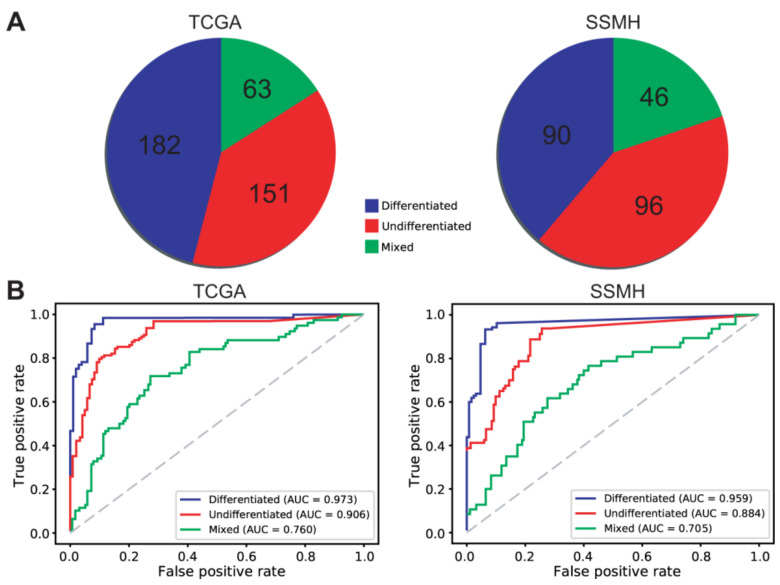
Slide-level classification results of the differentiated/undifferentiated classifier for the TCGA and SSMH datasets. (**A**) Pathologists’ classification for the TCGA and SSMH datasets. (**B**) The slide-level receiver operating characteristic (ROC) curves of the differentiated/undifferentiated classifier for the TCGA and SSMH datasets.

The two pathologists (S.H.L. and I.H.S.) identified 182 differentiated, 151 undifferentiated, and 63 mixed WSIs from the TCGA dataset and 90 differentiated, 96 undifferentiated, and 46 mixed WSIs from the SSMH dataset (Figure 6A). The ROC curves for the slide-level classification results of the differentiated/undifferentiated classifier are presented in Figure 6B.

## 4. Discussion

In the present study, DL-based classifiers were applied to discriminate the subclasses of GC tissues. We showed that this method can aid the quantitative evaluation of cancer tissues. This quantitative evaluation can be used in future studies for a deeper understanding of the prognostic and therapeutic significance of tumor subclass composition in GC. DL-based methods will be able to substitute the manual quantitative evaluation of the whole slides by pathologists, which is too laborious for routine application.

The differentiated/undifferentiated classifier yielded relatively poor performance compared to the normal/tumor and non-mucinous/mucinous classifiers. This result is surprising considering that the training of the differentiated/undifferentiated classifier had ten times more patches than the training of the non-mucinous/mucinous classifier. This result reflects the relative difficulty of the discrimination of the differentiated and undifferentiated cancer tissues. In some cases, discrimination between differentiated and undifferentiated tissues can be confusing [22]. Although the two gastrointestinal pathologists cooperated to make agreed annotations for the differentiated and undifferentiated cancer tissues, there will be grey areas that cannot be clearly discriminated because many mixed tumors have transition borders. The absence of a gold standard for differentiated and undifferentiated discrimination in the grey area was reflected in the DL-based classifier and the poorer performance of the differentiated/undifferentiated classifier. In fact, many misclassified patches came from the transition borders between the differentiated and undifferentiated cancer tissues, which were overlaid with a greenish color in the differentiated/undifferentiated probability heatmaps (Supplementary Figure S1). Therefore, the main discrimination power of the differentiated/undifferentiated classifier would be acceptable.

The histological type is known to be one of the most important factors in determining endoscopic treatment in EGC and chemotherapy for the advanced stage of GC. It is widely accepted that the risk of LNM is higher in patients with undifferentiated-type EGC than those with differentiated EGC, and therefore the indication criteria for endoscopic resection are more restricted in undifferentiated-type EGC [23,24]. Likewise, the histological type is a critical factor for the prediction of prognosis, recurrence patterns, and chemosensitivity in patients with advanced GC [25,26]. In addition, several recent studies demonstrated that mixed histology type in EGC is a risk factor for LNM [27,28,29]. Komatsu et al. showed that mixed histological type is itself an independent risk factor for LNM in EGC and that patients with mixed-type EGC showed significantly lower survival rates than patients with pure differentiated or undifferentiated types [28]. Furthermore, Lee et al. analyzed the relationship between the proportion of the undifferentiated-type components and LNM in EGC. They demonstrated that the presence of minor undifferentiated-type (>10% of total tumor volume) components should be considered when assessing the curative resection status of endoscopic resection for differentiated-type mucosal GC [29]. Moreover, Yuan et al. suggested that the clinical outcome of mucinous adenocarcinoma was far poorer than that of non-mucinous GC [30]. Given these results, the automatic classification of differentiated/undifferentiated and non-mucinous/mucinous tumor types and the calculation of the approximate composition ratio of each component is of great use for predicting the clinical outcomes of patients with GC.

GC is highly heterogeneous in both inter- and intra-tumor levels [31,32]. Studies for spatial tumor heterogeneity have been exploding in prevalence because of the emergence of molecular methods with high spatial specificity such as multi-region sequencing and single-cell sequencing [33,34]. However, a random sampling of tissues for these molecular tests would be inefficient. If candidate regions of molecular heterogeneity in a tissue slide could be identifiable before the tests, molecular testing could be more specific and efficient. DL-based tissue classifiers can help to investigate the spatial tumor heterogeneity by visualizing morphologically and molecularly heterogeneous regions in a tissue slide [35]. In previous studies, we could visualize the molecularly heterogeneous regions with different mutational or microsatellite instability status using DL-based classifiers [18,19]. In the present study, morphologically heterogeneous regions could also be discriminated. These DL methods can be used to determine the target regions for molecular tests to investigate the tumor heterogeneity more efficiently.

Although the DL-based classifiers showed promising applicability, there are some limitations. First of all, the black-box nature of DL limits the interpretability of DL models and remains a significant barrier in their validation and adoption in the clinic [36,37]. Recent efforts for explainable artificial intelligence will help to solve this problem [38]. In addition, the model should be validated with well-curated multi-national and multi-institutional datasets to secure generalizability. Currently, huge annotated datasets of digital pathology slides are not available for validation. However, many countries started to build nationwide datasets of tissue slides for various cancers. Therefore, the sizeable, well-curated data will soon be available for both the training and validation of DL models. Then, the performance and generalizability of DL-based tissue classifiers can be enhanced.

## 5. Conclusions

DL-based digital pathology tools have the potential to be integrated into routine pathology workflow to minimize subjectivity and enhance the accuracy of diagnosis and to provide more quantitative information on the tissue slides [39,40]. This information could help to improve our understanding of the prognostic value of tissue slides and the additional information will also support therapeutic decisions. Although there are still hurdles to overcome with the adoption of DL-based assistant tools, the accumulation of data and the evolution of DL algorithms may eventually open the era of computer-aided decision support for clinical practice.

## Data Availability

The TCGA data presented in this study are openly available in the GDC data portal (https://portal.gdc.cancer.gov/, accessed on 21 January 2021). The data from the Seoul St. Mary’s hospital are not publicly available due to privacy and ethical reasons.

## Supplementary Materials

The following are available online at https://www.mdpi.com/article/10.3390/cancers13153811/s1. Figure S1: The differentiated/undifferentiated probability heatmaps. Table S1: Summary of TCGA and SSMH data. Table S2: Classification results of the TCGA and SSMH datasets by the deep-learning-based classifiers.
