# Supplementary material for: Deep Learning for Automatic Subclassification of Gastric Carcinoma Using Whole-Slide Histopathology Images
Source: Cancers (Basel). 2021 Jul 29;13(15):3811. doi: 10.3390/cancers13153811 (PMC8345042; doi:10.3390/cancers13153811)
Supplement: Supplementary file 1 [file cancers-13-03811-s001.zip › cancers-1277591-sup-proof.pdf]

cancers

MDPI

Article

Supplementary Material: Deep Learning for Automatic Sub-classification of Gastric Carcinoma Using Whole-Slide Histopathology Images

Hyun-Jong Jang [1], In-Hye Song [2] and Sung-Hak Lee [2,*]

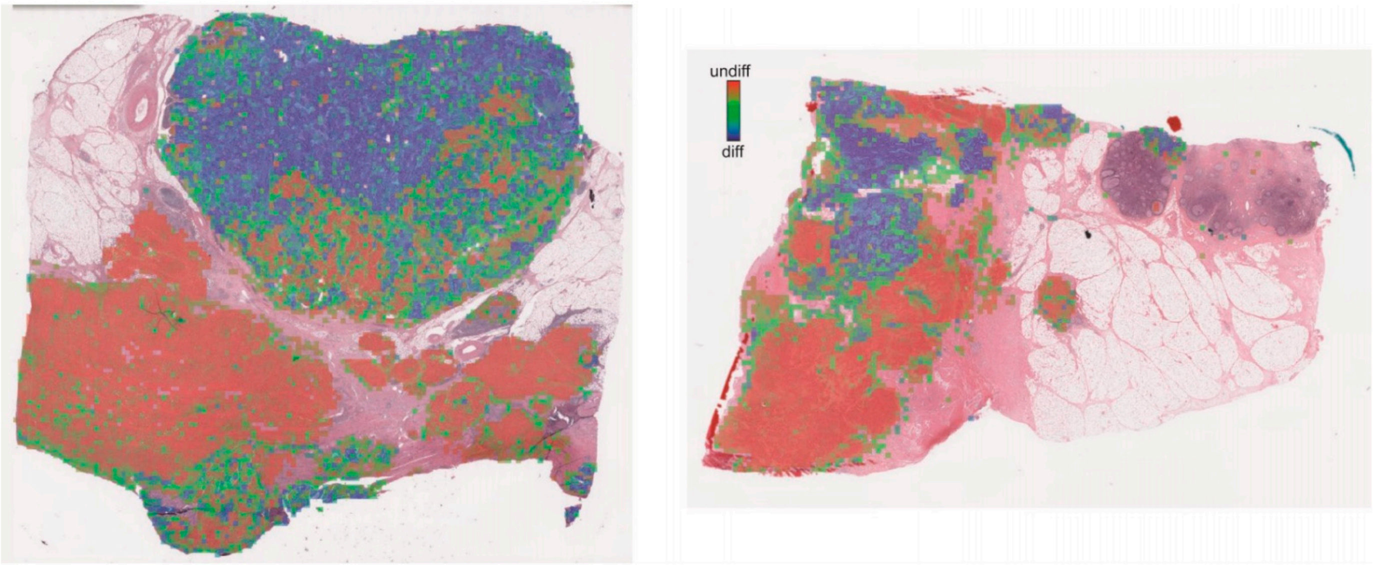

Figure S1. The differentiated/undifferentiated probability heatmaps. Representative gradient heatmaps demonstrating the relative differentiated/undifferentiated probability of the patches in the tissue slides. Insets: Color distribution between the differentiated (diff) and undifferentiated (undiff) probabilities.
